# Semi-supervised learning for genomic prediction of novel traits with small reference populations: an application to residual feed intake in dairy cattle

**DOI:** 10.1186/s12711-016-0262-5

**Published:** 2016-11-07

**Authors:** Chen Yao, Xiaojin Zhu, Kent A. Weigel

**Affiliations:** 1 Department of Dairy Science, University of Wisconsin, Madison, Madison, WI USA; 2 Department of Computer Science, University of Wisconsin, Madison, Madison, WI USA

## Abstract

**Background:**

Genomic prediction for novel traits, which can be costly and labor-intensive to measure, is often hampered by low accuracy due to the limited size of the reference population. As an option to improve prediction accuracy, we introduced a semi-supervised learning strategy known as the self-training model, and applied this method to genomic prediction of residual feed intake (RFI) in dairy cattle.

**Methods:**

We describe a self-training model that is wrapped around a support vector machine (SVM) algorithm, which enables it to use data from animals with and without measured phenotypes. Initially, a SVM model was trained using data from 792 animals with measured RFI phenotypes. Then, the resulting SVM was used to generate self-trained phenotypes for 3000 animals for which RFI measurements were not available. Finally, the SVM model was re-trained using data from up to 3792 animals, including those with measured and self-trained RFI phenotypes.

**Results:**

Incorporation of additional animals with self-trained phenotypes enhanced the accuracy of genomic predictions compared to that of predictions that were derived from the subset of animals with measured phenotypes. The optimal ratio of animals with self-trained phenotypes to animals with measured phenotypes (2.5, 2.0, and 1.8) and the maximum increase achieved in prediction accuracy measured as the correlation between predicted and actual RFI phenotypes (5.9, 4.1, and 2.4%) decreased as the size of the initial training set (300, 400, and 500 animals with measured phenotypes) increased. The optimal number of animals with self-trained phenotypes may be smaller when prediction accuracy is measured as the mean squared error rather than the correlation between predicted and actual RFI phenotypes.

**Conclusions:**

Our results demonstrate that semi-supervised learning models that incorporate self-trained phenotypes can achieve genomic prediction accuracies that are comparable to those obtained with models using larger training sets that include only animals with measured phenotypes. Semi-supervised learning can be helpful for genomic prediction of novel traits, such as RFI, for which the size of reference population is limited, in particular, when the animals to be predicted and the animals in the reference population originate from the same herd-environment.

## Background

When using whole-genome markers to predict breeding values or future phenotypes, a main challenge is the construction of an effective reference population that includes genotyped and phenotyped individuals for training the prediction model. Both size of the reference population and genetic distance between the reference population and the current pool of selection candidates are critical factors [1, 2]. Given a sufficient number of individuals in the reference population or training set, there are several highly effective “supervised learning” techniques for training prediction models, such as Bayesian regression models, genomic best linear unbiased prediction (BLUP), kernel-based methods, and machine-learning algorithms (e.g., [3–6]). Very large reference populations are available for some traits and species, such as for milk yield in Holstein dairy cattle, but for other traits such as feed efficiency, the size of the reference population is often limited by the exorbitant cost or intensive labor requirements associated with measuring individual phenotypes. For dry matter intake (DMI) in dairy cattle, researchers have collated data from multiple countries to estimate genetic parameters and perform genomic prediction [7, 8]. Alternatively, after several years of genomic selection on traits such as milk yield, hundreds of thousands of dairy cows and bulls have been genotyped, and useful information that can enhance the accuracy of genomic prediction for feed efficiency could be extracted from the available genomic data of animals that have not been measured for the phenotype of interest.

A powerful tool from the machine-learning community has potential for addressing this challenge, i.e. a technique known as semi-supervised learning. As its name suggests, semi-supervised learning refers to models that combine attributes of supervised and unsupervised learning [9]. Most genomic prediction models that are currently popular, such as genomic BLUP and Bayesian regression, rely on supervised model training. In supervised models, a measured phenotype is provided as the desired “label” for an animal, and this phenotype supervises the process of model training to predict future phenotypes from the corresponding genotypes. In contrast, for unsupervised learning no measured phenotype is available to supervise the labeling of an animal’s genotype. An example of unsupervised learning is the use of principal component analysis to cluster animals into groups based on the similarity of their genotypes.

In this study, and in the context of genomic selection for enhanced feed efficiency in dairy cattle, we applied a simple but widely used semi-supervised learning algorithm known as the “self-training” model [9]. During the learning process, this model uses its own predictions that are derived from the subset of “labeled” individuals with measured phenotypes, to teach itself on the relationships between genotypes and phenotypes of “unlabeled” individuals with missing phenotypes. The self-training model that can be wrapped around a wide variety of genomic prediction methods. In this study, we extended a typical machine-learning genomic selection model, namely the support vector machine (SVM) [10, 11], which provided higher prediction accuracies of residual feed intake (RFI) using whole-genome molecular markers than the random forests model [12]. In this approach, the training data consist of a combination of individuals with measured phenotypes and model-derived “self-trained” phenotypes, and both sources of data are used for subsequent prediction of genomic breeding values for RFI. Knowledge about the genomic relationships within the population of animals without phenotypes can contribute to the accuracy of selection, and if this is successful, the resulting prediction accuracy will exceed that of a supervised learner trained with only the animals that have measured phenotypes. The underlying assumptions are that the measurement of additional phenotypes for the novel trait is difficult or expensive, and that additional genotypes of animals without novel trait phenotypes are available at little or no cost.

Although, until now, self-training has not been introduced in an animal breeding context, it has been used for a variety of promising applications in the broader subject area of artificial intelligence. For example, Rosenberg et al. [13] implemented a semi-supervised learning approach as a wrapper around an existing object detector and achieved results that were comparable to a model trained with a much larger set of fully labeled data. McClosky et al. [14] self-trained an effective two-phase parser-re-ranker system using unlabeled data, and the semi-supervised model achieved a 12% reduction in error compared to the best previous result for parsing. Tang et al. [15] proposed the semi-supervised transductive regression forest for real-time articulated hand pose estimation and showed that accuracies could be improved by considering unlabeled data. In genetics, this method has been used to increase the accuracy of gene start prediction, by combining models of protein-coding and non-coding regions and models of regulatory sites near the gene start [16]. In the area of gene identification, a self-training model was used to find genes in eukaryotic genomes in parallel with statistical model estimates that were taken directly from anonymous genomic DNA [17].

In this work, we present the first application of a self-training algorithm in the context of genomic selection of livestock, using RFI as the phenotype of choice for measuring feed efficiency in dairy cattle. A comprehensive evaluation of the model training process was undertaken in order to facilitate effective application of semi-supervised learning techniques to predict a complex trait such as RFI using whole-genome molecular markers. This study focused on determining how performance of the predictor is affected by the number of “labeled” individuals with measured phenotypes and “unlabeled” individuals with self-trained phenotypes. It also aimed at providing new insights in genomic selection by enhancing prediction accuracy through the inclusion of animals without measured phenotypes via semi-supervised learning methods.

## Methods

### Description of the semi-supervised self-training algorithm

**Fig. 1:**
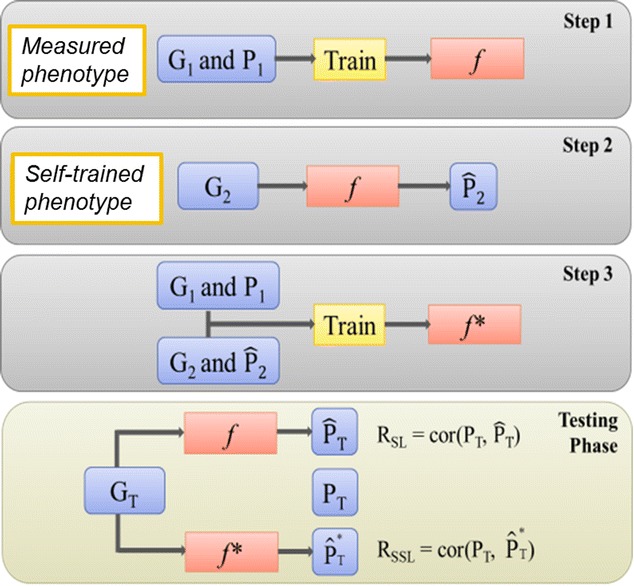
Illustration of the self-training algorithm. Step 1: train a base predictor, $f$, using ${\text{G}}_{1}$ and ${\text{P}}_{1}$ from animals with measured phenotypes. Step 2: predict self-trained phenotypes, ${\hat{\text{P}}}_{2}$, based on ${\text{G}}_{2}$ for animals without measured phenotypes. Step 3: combine ${\text{G}}_{1}$, ${\text{G}}_{2}$, ${\text{P}}_{1}$, and ${\hat{\text{P}}}_{2}$ to train a new predictor, $f^{*}$. In the testing phase, compare accuracies of $f$ and $f^{*}$ on the testing set (${\text{R}}_{\text{SL}}$ and ${\text{R}}_{\text{SSL}}$). First, predict phenotypes ${\hat{\text{P}}}_{\text{T}}$ and ${\hat{\text{P}}}_{\text{T}}^{ *}$ based on ${\text{G}}_{\text{T}}$ using $f$ and $f^{*}$, respectively and second, calculate ${\text{R}}_{\text{SL}}$ (${\text{R}}_{\text{SSL}}$) as the correlation between ${\hat{\text{P}}}_{\text{T}}$ (${\hat{\text{P}}}_{\text{T}}^{ *}$) and ${\text{P}}_{\text{T}}$

One particular semi-supervised learning strategy, i.e. the self-training model, was used in this study. Animals with measured phenotypes were separated into training and testing sets. The training and testing sets included animals with genotypes, ${\text{G}}_{1}$ and ${\text{G}}_{\text{T}}$, and measured phenotypes, ${\text{P}}_{1}$ and ${\text{P}}_{\text{T}}$. Animals without measured phenotypes only contributed genotypes, denoted as ${\text{G}}_{2}$. During self-training, an initial model was trained using the training set of ${\text{G}}_{1}$ and ${\text{P}}_{1}$ to formulate the base predictor ($f$). This predictor was then used to predict the self-trained phenotypes, ${\hat{\text{P}}}_{2}$, for the individuals that lacked measured phenotypes. Next, the individuals comprising ${\text{G}}_{2}$ and ${\hat{\text{P}}}_{2}$ were added to the training set in order to train a new predictor ($f^{*}$). In the testing phase, accuracies of $f$ and $f^{*}$ within the testing set (denoted as ${\text{R}}_{\text{SL}}$ and ${\text{R}}_{\text{SSL}}$) were compared. First, phenotypes ${\hat{\text{P}}}_{\text{T}}$ and ${\hat{\text{P}}}_{\text{T}}^{ *}$ were predicted from ${\text{G}}_{\text{T}}$ using $f$ and $f^{*}$, respectively. Second, ${\text{R}}_{\text{SL}}$ (${\text{R}}_{\text{SSL}} )$ was computed as the correlation between ${\hat{\text{P}}}_{\text{T}}$ (${\hat{\text{P}}}_{\text{T}}^{ *}$) and ${\text{P}}_{\text{T}}$. The self-training algorithm is summarized below and illustrated in Fig. 1.

*Step 1*: Train a base predictor, $f$, using genotypes ${\text{G}}_{1}$ and phenotypes ${\text{P}}_{1}$ from animals with measured phenotypes.

*Step 2*: Predict the self-trained phenotypes (${\hat{\text{P}}}_{2}$) from the genotypes ${\text{G}}_{2}$ for animals without measured phenotypes.

*Step 3*: Combine genotypes ${\text{G}}_{1}$ and ${\text{G}}_{2}$ with phenotypes ${\text{P}}_{1}$ and ${\hat{\text{P}}}_{2}$, in order to train a new predictor, $f^{*}$, which is used to compute the final genomic predictions.

An SVM algorithm was used as the genomic prediction model and implemented using the “svm” function of the “e1071” package Version 1.6-1 in R [18] with radial basis kernel and default parameters tuned within the training set.

### Definition of the RFI phenotypes

The data consisted of 792 lactating Holstein dairy cows from the Allenstein Dairy Herd at the University of Wisconsin–Madison. Phenotypes used in this study were a subset of the data analyzed by Tempelman et al. [19], in which a detailed description of the data is provided. All animals with measured phenotypes were recorded for daily DMI, daily milk yield, weekly milk composition (fat %, protein %, and lactose %), and weekly body weight (BW) during the period from 50 to 200 days postpartum. Quality control and editing were similar to Yao et al. [12]. Only one lactation record per animal was used.

Residual feed intake was defined as the deviation of an animal’s feed intake from the average intake of its cohort, after adjusting for milk production and composition, maintenance of body weight, and known environmental differences. Fixed environmental effects included year-season of calving (YSC) with 19 levels (year: 2007–2013; season: January–March, April–June, July–September, or October–December) and parity-by-age at calving (ParAge) with 20 levels (1st parity: ≤23, 24, 25, 26, or ≥27 months; 2nd parity: ≤35, 36, …, 40, or ≥41 months; 3rd parity or later: ≤48, 49,50, 51, 52–56, 57–61, 62–69, or ≥70 months), while medium days in milk for the week (dim) was included as a covariate. A total of 81 rations used in specific nutrition experiments were modeled as random cohort effects. Weekly mean RFI phenotypes were the residuals from this model, calculated as:

$$ {\mathbf{y}} = \mu + {\mathbf{YSC}} + {\mathbf{ParAge}} +\varvec{\beta}_{1} {\mathbf{dim}} +\varvec{\beta}_{2} {\mathbf{MilkE}} +\varvec{\beta}_{3} {\mathbf{MBW}} + {\mathbf{ration}} + {\mathbf{RFI}}, $$

where ${\mathbf{y}}$ is a vector of DMI phenotypes, $\mu$ is the population mean, ${\mathbf{YSC}}$ is a vector of the fixed effects of year-season of calving, ${\mathbf{ParAge}}$ is a vector of fixed effects of parity-by-age at calving interaction, ${\mathbf{dim}}$ is a vector of the fixed covariate of the medium days in milk during the week with regression coefficient $\varvec{\beta}_{1}$, ${\mathbf{MilkE}}$ is a vector of the fixed covariate of energy expressed in milk (defined in [12]) with regression coefficient $\varvec{\beta}_{2}$, ${\mathbf{MBW}}$ is a vector of the fixed covariate of the metabolic BW (i.e., BW^0.75^) with regression coefficient $\varvec{\beta}_{3}$, ${\mathbf{ration}}\,\sim\,N\left( {0,{\mathbf{I}}\upsigma_{r}^{2} } \right)$ is a vector of the random cohort effects, and ${\mathbf{RFI}}\,\sim\,N\left( {0,{\mathbf{I}}\upsigma_{e}^{2} } \right)$ is a vector of random residuals. The mixed model equations were solved with the restricted maximum likelihood (REML) method using the R-package “lme4”, Version 0.999999-0 [14]. Then, for each animal, the RFI phenotype was set to the mean of weekly RFI estimates available for that animal.

### Genetic marker data

Single nucleotide polymorphism (SNP) genotypes were available for the 792 cows with measured RFI phenotypes and for 3000 cows without measured RFI phenotypes; these 3000 cows included 1127 Holstein cows from four other university research herds (University of Florida, Iowa State University, Michigan State University, and Virginia Tech University) and 1813 Holstein cows born in 2009 and selected randomly from the Council on Dairy Cattle Breeding (CDCB; Bowie, MD) Holstein genotype database. Raw genotypes represented various low-density and medium-density arrays, and missing genotypes were imputed to higher density using genotype information from bulls and cows in the CDCB database [20, 21]. Any remaining missing genotypes (about 2%) were imputed by using rounded allele frequencies from the current US Holstein population. SNPs with minor allele frequencies lower than 5% were removed. A total of 57,491 SNPs per individual were available for the genomic prediction analysis. The SNP genotype at each locus was coded as 0, 1, or 2, counting the number of copies of the minor allele.

### Design of the validation study

In practical applications of genomic selection in dairy cattle, training is typically carried out using historical animals, and the target population of selection candidates for genomic prediction includes, but is not limited to, offspring and descendants of animals in the training set [22]. In this study, genomic prediction models were trained using 540 animals born before January 1, 2010 and validation of the prediction accuracies was carried out using 252 animals born after January 1, 2010. In order to assess the impact of size of the reference population on the accuracy of genomic prediction with supervised learning, size of the training set was varied from 20 to 540 in increments of 20 (i.e., $n$ = 20, 40, 60, …, 540), and prediction accuracy for a given $n$ was averaged over 100 random samples from the training set. To assess the impact of the relative numbers of animals with measured phenotypes versus self-trained phenotypes in the training set, the number of animals with measured phenotypes was set to 300, 400, or 500, whereas the number of animals with self-trained phenotypes was set to 200, 400, 600, or 800. Again, the accuracy of genomic prediction was evaluated by averaging over 100 replicates of each scenario, for which reference animals with and without measured RFI phenotypes were sampled from the respective pools of available animals.

## Results and discussion

### Prediction accuracy of supervised learning

**Fig. 2:**
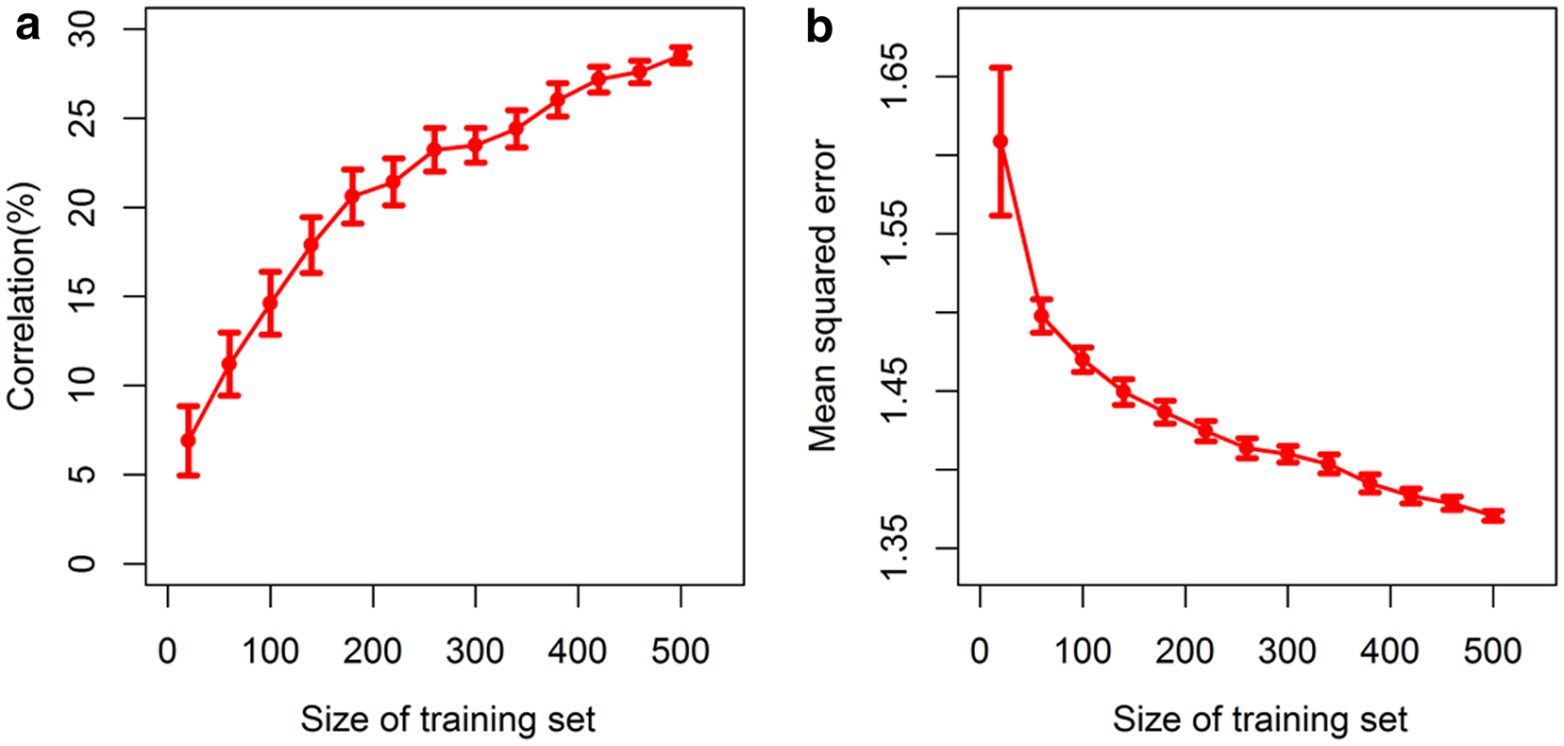
Average accuracy of genomic prediction for 252 animals in the testing set. Accuracy of genomic prediction was measured as the **a** correlation or **b** mean squared error between predicted and measured residual feed intake phenotypes, plotted against the size of the training set of animals with measured phenotypes. The results for each training set size were based on 100 replicates using $n$ = 20, 60, …, 500 random samples from the full training set of 540 individuals with measured phenotypes. *Error bars* indicate the 95% confidence interval for each mean

The goal of semi-supervised learning in the context of genomic prediction for novel traits is to train a learner using genotyped animals that may or may not have a measured phenotype for the trait of interest. First, we characterized the sensitivity of the prediction model for supervised learning to the size of the training set, in order to assess the possible gains in prediction accuracy that could be achieved by adding unlabeled animals without measured phenotypes. If performance of the model is already at its maximum, given a training set with measured phenotypes of a specific size, then gains by including additional animals without measured phenotypes are unlikely. Accuracies of prediction for supervised learning with training sets that range in size from 20 to 540 animals are in Fig. 2, where each point represents the correlation (Fig. 2a) and mean squared error (MSE) (Fig. 2b) between genomic predictions and actual RFI phenotypes for cows in the testing set, averaged over 100 replicates. As shown in Fig. 2, accuracy measured as the correlation increased and MSE decreased rapidly until the training set reached about 200 animals, and accuracy changed more slowly thereafter, while 95% of the confidence intervals decreased steadily as the size of the training set increased. Clearly a reference population with less than 200 individuals with measured phenotypes was not sufficient to train the prediction model. The reduction in standard error of the predictive accuracy may be attributed to a greater likelihood that the same individuals would be repeated among different replicates as size of the training set increased. Overall, Fig. 2 shows that the accuracy of genomic prediction increased throughout the range of training sets considered, and therefore opportunities for improvement exist by including animals with self-trained phenotypes. Based on these results, we set the size of the training set with measured phenotypes to 300, 400, and 500 for the subsequent semi-supervised learning analyses.

### Prediction accuracy of semi-supervised learning

**Fig. 3:**
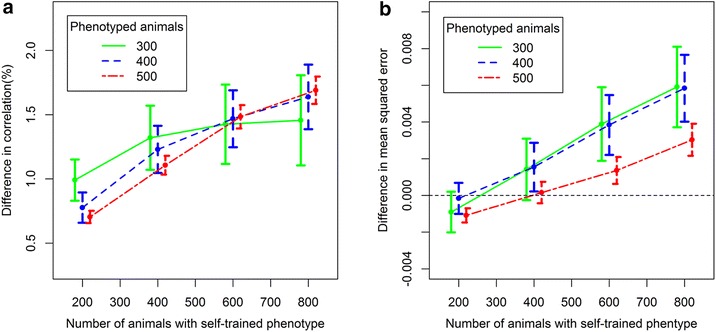
Difference in accuracies of genomic predictions between the supervised and self-training algorithms. Accuracy of genomic prediction was tested for 252 animals in the testing set, and measured as the **a** correlation or **b** mean squared error between predicted and measured residual feed intake phenotypes. The number of initial reference animals with measured phenotypes was 300, 400, or 500, and a total of 200, 400, 600, and 800 animals with self-trained phenotypes were added to the training set using the self-training algorithm. *Error bars* indicate the 95% confidence intervals for each mean of 100 replicates

Second, we assessed how the number and proportion of animals with self-trained phenotypes changed the accuracy of genomic predictions for animals in the testing set. This change was calculated by subtracting the accuracy of the initial supervised learning model from the final accuracy of the corresponding semi-supervised learning model, i.e. ${\text{R}}_{\text{SL}} - {\text{R}}_{\text{SSL}}$. The average baseline accuracies, measured as the correlation (as the MSE) between predicted and measured RFI phenotypes for supervised SVM models trained with 300, 400, and 500 animals with measured phenotypes were equal to 22.9 (1.41), 27.0 (1.39), and 28.7% (1.37), respectively. The results in Fig. 3a suggest that including animals with self-trained phenotypes can improve prediction accuracy and the degree of improvement tended to increase as the number of additional animals with self-trained phenotypes increased. In addition, a 0% change was not included in any of the 95% confidence intervals in the graph, which suggests that results from semi-supervised and supervised learning models were significantly different. The MSE in Fig. 3b improved when adding 200 animals with self-trained phenotypes and started to decrease at 400 animals with self-trained phenotypes. Although the increases in Fig. 3a were only up to 1.7%, these increases are valuable for traits with an estimated heritability of 0.15 [19]. As shown by Pryce et al. [23], the accuracy (correlation) of genomic prediction of RFI only increased by 2% (from 11 to 13%) when adding 939 heifers from New Zealand to the reference population of 843 Australian heifers. However, the cost to measure the individual feed intake of these 939 extra heifers was not negligible.

Figure 3a also indicates that, for a given number of animals with self-trained phenotypes, the improvement in accuracy measured as the correlation due to semi-supervised learning depends on the number of animals with measured phenotypes. The benefit of adding a given number of animals with self-trained phenotypes was greater for models that included fewer animals with measured phenotypes. A similar outcome was reported by Filipovych et al. [24] when using semi-supervised learning to classify brain images of patients with uncertain diagnoses. The reason is that, in general, semi-supervised learning is used to address situations in which labeled data are scarce [9]. In other words, if the sample of training animals is too small or does not completely represent the genomes of animals in the testing set, over-fitting specific nuances of the training set animals may lead to poor generalization to future testing populations. In semi-supervised learning, the extra genomic information from animals without measured phenotypes may help to reduce the chance of over-fitting. Therefore, potential uses of semi-supervised learning in animal breeding could focus on traits such as RFI, for which the number of reference animals with phenotypes is small. When the number of animals with measured phenotypes is sufficient, the potential gains by adding information from animals with self-trained phenotypes is limited. However, this trend was not clear for the MSE shown in Fig. 3b. Figure 3a also shows that the slopes of all three curves decreased as the number of animals with self-trained phenotypes increased. In other words, the gain in prediction accuracy (correlation) associated with an increase from 200 to 400 animals with self-trained phenotypes was greater than the gain in accuracy associated with an increase from 400 to 600, and so on. Therefore, we may approach a plateau beyond which inclusion of more animals with self-trained phenotypes provides no additional benefit.

**Fig. 4:**
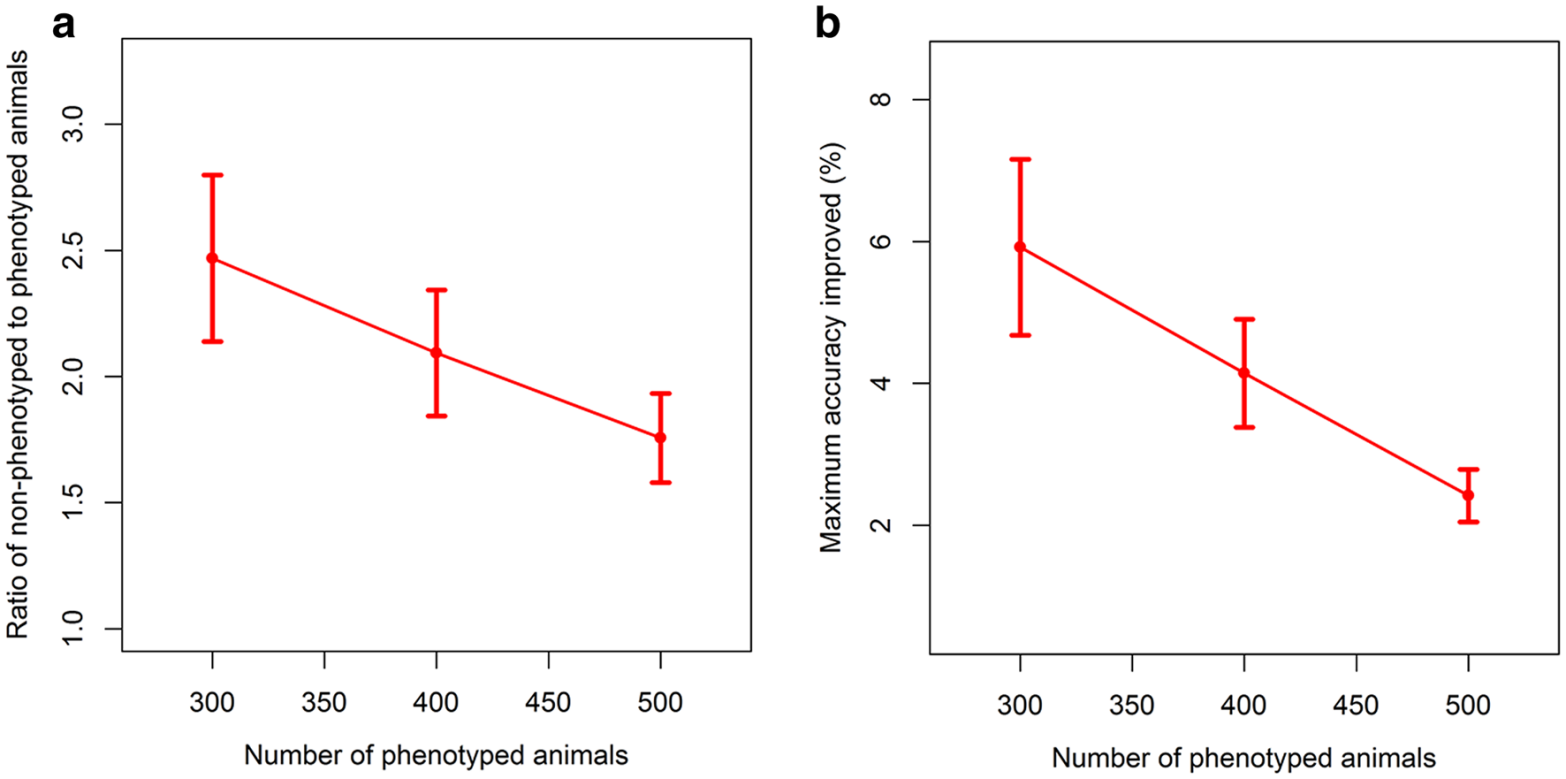
**a** Ratio of the number of animals with self-trained phenotypes to that with measured phenotypes and **b** maximum improvement of the correlation (%). Incremental improvement in accuracy of prediction measured as the correlation between predicted and actual residual feed intake phenotypes within a replicate due to additional animals with self-trained phenotypes was smaller than 0.01%. The numbers of animals with measured phenotypes were 300, 400, and 500, whereas the numbers of animals with self-trained phenotypes started with 200 and increased in increments of 200 additional animals. *Error bars* indicate the 95% confidence intervals for means of 100 replicates

Third, we evaluated the number of animals with self-trained phenotypes that must be added to the training set of animals with measured phenotypes in order to achieve the maximum improvement in prediction accuracy measured using as the correlation. For this purpose, we fixed the number of animals with measured phenotypes, and we increased the number of animals with self-trained phenotypes by 200, until we reached the point at which additional improvements in accuracy were less than 0.01% for a given replicate. Figure 4a shows the ratio of the number of animals with self-trained phenotypes to the number of animals with measured phenotypes at the point at which improvements in accuracy measured as the correlation became negligible, for a given size of the initial training set. These results indicate that the optimal ratio of animals with self-trained to animals with measured phenotypes decreases as the size of the initial labeled training set increases and thus, that more animals with self-trained phenotypes are needed to compensate for the smaller labeled training sets. The highest average accuracies (correlations) achieved by semi-supervised learning were equal to 30.9, 31.4, and 30.9% for initial training sets of 300, 400, and 500 animals with measured phenotypes, respectively. Figure 4b shows that the maximum improvement achieved in prediction accuracy measured as the correlation was equal to 5.9, 4.1, and 2.4% by including animals with self-trained phenotypes in relation to the size of the initial labeled training set. Results show that maximum gains in prediction accuracy decreased as the size of the initial training set increased. In each case, these exceeded the correlation of 29.2% that was achieved using supervised learning with the full training set of 540 animals with measured phenotypes. Thus, in this study, self-training models achieved prediction accuracies measured as correlations that were comparable to those obtained from fully supervised models that were trained using larger reference populations with measured phenotypes.

Prediction accuracy measured as the correlation is an estimator of the linear relationship between predictions and responses and does not address the bias of predictions, in contrast to the MSE, as noted by González-Recio et al. [5]. The optimal number of animals with self-trained phenotypes based on minimizing MSE, was less than 400, as shown in Fig. 3b. This optimal number was smaller than when it was estimated by using the correlation based on minimizing the correlation. Thus, depending on the purpose and criterion used for the accuracy of the prediction, the optimal number of animals with self-trained phenotypes may differ. If only the linear relationship is important, it is likely that a larger number of animals with self-trained phenotypes could be used compared with a situation where the bias of the predictions is critical.

### Advantages and limitations of self-training models

The major advantages of self-training models for semi-supervised learning are threefold. First, implementation of the algorithm is simple. Second, self-training can be wrapped around any prediction model, such as SVM, genomic BLUP, or Bayesian regression. Third, if additional genotypes of animals without phenotypes are readily available (which is almost always the case), the additional costs are negligible. However, a potential disadvantage is that a self-training model is allowed to learn from its own predictions. Thus, a mistake that is made early in the learning process may reinforce itself in the self-trained phenotypes, and this could lead to poorer predictions from the final genomic prediction model. Such mistakes can occur, for example, if assumptions in the prediction model are inappropriate for a given dataset, or if the number of animals with measured phenotypes in the first step is inadequate to construct a useful predictor. As an example, we explored the use of a random forests model with a set-up that is similar to that in Yao et al. [12] instead of using SVM in the self-training model, and found no improvement from using self-training model. As shown in [12], SVM resulted in substantially better prediction accuracy (correlation) than that obtained with the random forests model when predicting RFI (20.5 vs. 8.876%). Therefore, choosing an appropriate prediction model for the self-training method is essential. Various heuristics have been introduced to address this problem [9], and, in practice, many researchers have noted that semi-supervised learning does not always improve the accuracy of prediction or classification [25, 26]. Therefore, additional studies in animal breeding or livestock production beyond this initial application to genomic prediction of RFI in Holstein dairy cattle are needed.

## Conclusions

We introduced a self-training model, chosen from the class of semi-supervised learning strategies, as a novel method to achieve potential improvements in the accuracy of genomic prediction, with a specific application to RFI in dairy cattle. Our results suggest that a self-training algorithm wrapped around a SVM prediction model may increase the accuracy of genomic prediction by collecting additional genomic information about the population from animals without measured phenotypes. For a given training set of animals with measured phenotypes, improvements in prediction accuracy measured as the correlation associated with semi-supervised learning increased as the number of additional animals with self-trained phenotypes increased, and eventually reached a plateau. In addition, improvements in accuracy measured as the correlation between predicted and measured RFI phenotypes from adding animals with self-trained phenotypes to the reference population were smaller when more animals with measured phenotypes were available in the initial testing set. The optimal number of animals with self-trained phenotypes can be smaller when predictions are evaluated based on MSE, rather than based on a correlation. Semi-supervised learning may be helpful to enhance the accuracy of genomic prediction for novel traits that are difficult or expensive to measure, and hence for small reference populations, but potential gains for other traits beyond RFI in dairy cattle should be studied.
